# Na^+^/H^+^ exchanger 1 has tumor suppressive activity and prognostic value in esophageal squamous cell carcinoma

**DOI:** 10.18632/oncotarget.13645

**Published:** 2016-11-26

**Authors:** Yosuke Ariyoshi, Atsushi Shiozaki, Daisuke Ichikawa, Hiroki Shimizu, Toshiyuki Kosuga, Hirotaka Konishi, Shuhei Komatsu, Hitoshi Fujiwara, Kazuma Okamoto, Mitsuo Kishimoto, Yoshinori Marunaka, Eigo Otsuji

**Affiliations:** ^1^ Division of Digestive Surgery, Department of Surgery, Kyoto Prefectural University of Medicine, Kyoto 602-8566, Japan; ^2^ Department of Pathology, Kyoto Prefectural University of Medicine, Kyoto 602-8566, Japan; ^3^ Department of Molecular Cell Physiology and Bio-Ionomics, Graduate School of Medical Science,Kyoto Prefectural University of Medicine, Kyoto 602-8566, Japan; ^4^ Japan Institute for Food Education and Health, St. Agnes’ University, Kyoto 602-8013, Japan

**Keywords:** esophageal squamous cell carcinoma, Na^+^/H^+^ exchanger 1, epithelial-mesenchymal transition, prognosis, Notch signaling

## Abstract

Na^+^/H^+^ exchanger 1 (NHE1) is a plasma membrane transporter that controls intracellular pH and regulates apoptosis and invasion in various cancer cells. However, the function of NHE1 in esophageal squamous cell carcinoma (ESCC) cells and the relationship between the expression of NHE1 and prognosis of ESCC remain unclear. We found that the knockdown of NHE1 in ESCC cells inhibited apoptosis and promoted cell proliferation, migration, and invasion and showed increases in Snail, β-catenin, and activation of PI3K-AKT signaling, which was consistent with the results obtained from microarrays. Microarrays results suggested that the knockdown of NHE1 suppressed Notch signaling pathway. An immunohistochemical investigation of 61 primary ESCC samples revealed that NHE1 was expressed at higher levels in well-differentiated tumors. The 5-year survival rate was poorer in the NHE1 low group (57.0%) than in the NHE1 high group (82.8%). Multivariate analyses revealed that the weak expression of NHE1 was associated with shorter postoperative survival (hazard ratio 3.570, 95% CI 1.291-11.484, *p* = 0.0135).We herein demonstrated that the suppression of NHE1 in ESCC may enhance malignant potential by mediating PI3K-AKT signaling and EMT via Notch signaling, and may be related to a poor prognosis in patients with ESCC.

## INTRODUCTION

Ion transport and cytoplasmic pH play crucial roles in multiple cell functions including the control of cell volume, cell growth and proliferation, growth factor activity, invasion, oncogenesis and malignant transformation [1–6]. In the process of caner metastasis, extracellular pH, local ion concentrations and water transport are also known to be coordinately regulated with the release of cell adhesion contacts, controlled cytoskeletal dynamics, and the digestion and reorganization of the extracellular matrix [5, 7].

Na^+^/H^+^ exchanger 1 (NHE1) is a transmembrane transporter that is ubiquitously expressed in all organisms [8–11]. To date, 10 isoforms have been identified in the human NHE family [9, 12, 13]. NHE1 regulates intracellular pH and cell volume by removing a proton in exchange for an extracellular sodium ion, and affects cell growth, proliferation, migration, and apoptosis [2, 9, 10, 12, 14]. Cancer cells have more alkaline intracellular pH and acidic extracellular pH values than normal cells due to the activation of NHE1 [1, 6, 15]. NHE1 activity is stimulated the interactions of the C-terminal tail with intracellular proteins, lipids and signal transduction pathway [14, 16–18].

Recent studies revealed that NHE1 plays important roles in various cancers such as breast cancer [18, 19], hepatocellular carcinoma [20], colon cancer [21], pancreatic ductal adenocarcinoma [22], prostate cancer [23], cervical cancer [24], and neuroblastoma [25, 26]. Chiang et al. showed that epidermal growth factor upregulated the expression of NHE1 and promoted cervical cancer cell invasiveness, and high expression level of NHE1 was associated with poor clinical outcomes in cervical cancer [24]. In breast cancer, CD44 increased cell invasion and activated MAPK signaling pathway through promotion of the expression of NHE1, and the repression of NHE1 by PPARγ ligands sensitized tumor cells to paclitaxel [18, 19]. However, the role of NHE1 in esophageal squamous cell carcinoma (ESCC) cells remains uncertain. Furthermore, the clinicopathological meaning of the expression of NHE1 in human ESCCs has not yet been evaluated.

Therefore, the objective of the present study was to investigate the role of NHE1 in the cell proliferation, apoptosis, migration and invasion of ESCC. A microarray analysis showed that the expression levels of many genes related to tumor growth, apoptosis, epithelial-mesenchymal transition (EMT), and Notch signaling were altered in cells transfected with NHE1 siRNA. Furthermore, we analyzed the expression of NHE1 in human ESCC samples and determined its relationships with the clinicopathological features and prognoses of ESCC patients. Our results revealed the important role of NHE1 in tumor progression in ESCC.

## RESULTS

### NHE1 controls cell proliferation and apoptosis in ESCC cells

**Figure 1:**
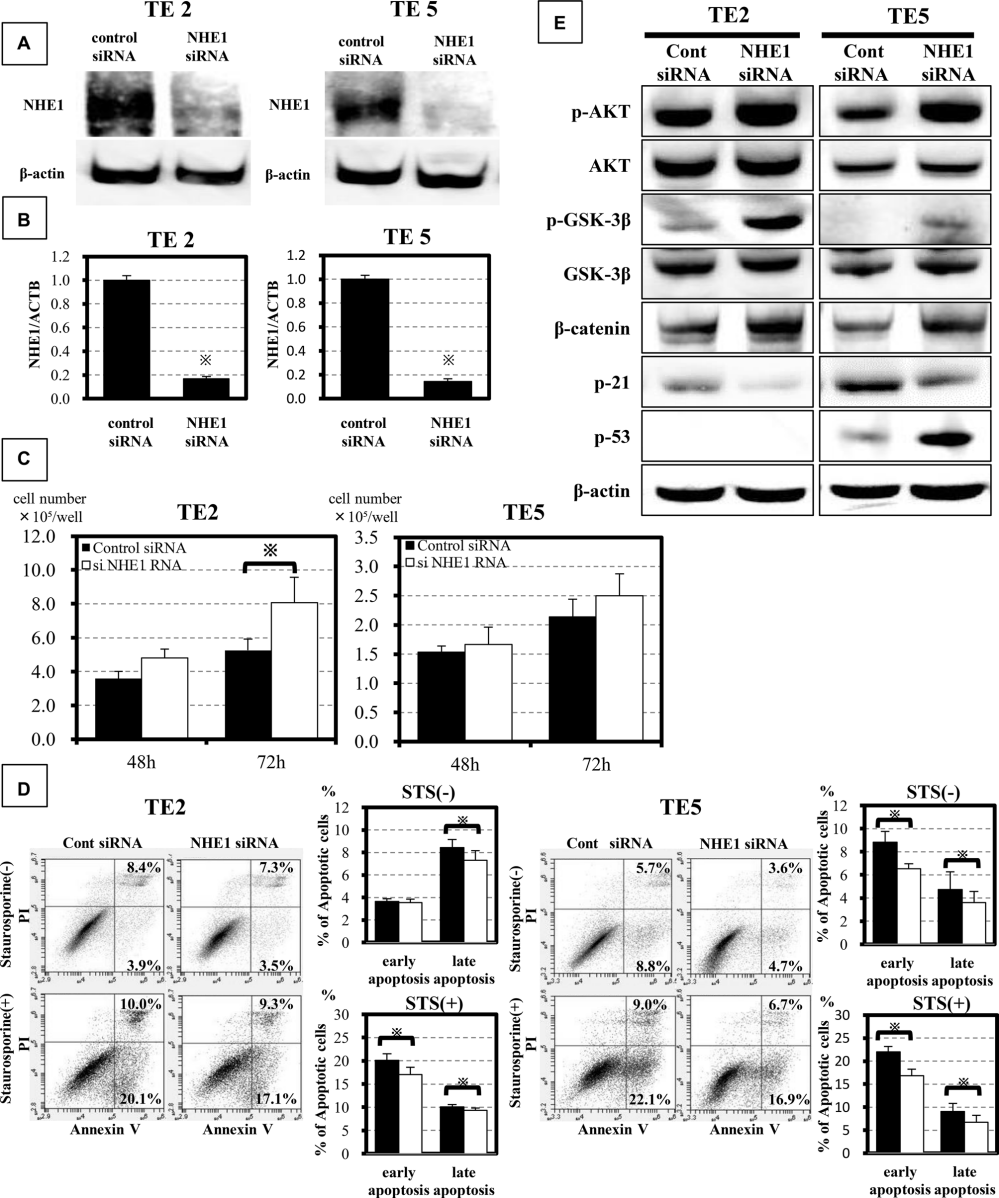
NHE1 controlled proliferation and apoptosis in ESCC cells via the PI3K-AKT pathway (**A**) NHE1 siRNA significantly inhibited the expression of the NHE1 protein. (**B**) NHE1 siRNA effectively reduced NHE1 mRNA levels in TE2 and TE5 cells. Mean ± SEM; *n* = 3. *P < 0.001 significantly different from the control siRNA group. (**C**) The down-regulation of NHE1 accelerated the proliferation of TE2 and TE5 cells. The number of cells was counted 48 and 72 h after siRNA transfection. Mean ± SEM; *n* = 6. *P < 0.05 significantly different from the control siRNA group. (**D**) The down-regulation of NHE1 reduced spontaneous and induced cell death in TE2 and TE5 cells. Cells transfected with control or NHE1 siRNA were treated with staurosporine (200 nmol/L) for 24 h. Mean ± SEM. *n* = 6. *P < 0.05 significantly different from the control siRNA group. (**E**) Detection of the phosphorylation of AKT, glycogen synthase kinase-3β (GSK-3β), β-catenin, p21and p53 in NHE1-knockdown TE2 and TE5 cells. NHE1 activated PI3K-AKT signaling.

We conducted knockdown experiments with NHE1 siRNA in TE2 and TE5 cells in order to determine its effects on cell proliferation, apoptosis, and PI3K-AKT signaling. In both cell lines, NHE1 siRNA effectively reduced NHE1 protein (Figure 1A) and mRNA levels (Figure 1B). TE2 cell counts 72 h after siRNA transfection were significantly higher in NHE1 siRNA-transfected cells than in control cells (Figure 1C). In TE5 cells, cell proliferation was greater in NHE1 siRNA-transfected cells than in control cells (Figure 1C). In order to determine the role of NHE1 in ESCC cell survival, we analyzed apoptosis in TE2 cells and TE5 cells with NHE1 siRNA. The down-regulation of NHE1 decreased early apoptosis in TE5 cells and late apoptosis in TE2 and TE5 cells 48 h after siRNA transfection (Figure 1D). Furthermore, NHE1 siRNA decreased staurosporine stimulus-induced early apoptosis and late apoptosis in both cell lines (Figure 1D). These results indicate that suppression of NHE1 expression promote cell proliferation and inhibit apoptosis. We also conducted overexpression study. Cells transfected Control-HaloTag^®^ plasmid and NHE1-HaloTag^®^ plasmid were stained in red (Supplementary Figure S1A) and NHE1-HaloTag^®^ plasmid increased NHE1 mRNA levels (Supplementary Figure S1B). NHE1 overexpression in TE2 cells and TE5 cells inhibited cell growth (Supplementary Figure S1C) and induced apoptosis (Supplementary Figure S1D) as opposed to knockdown of NHE1.Furthermore, we examined the effects of the down-regulation of NHE1 on PI3K-AKT signaling. A Western blot analysis (Figure 1E) showed that the down-regulation of NHE1 increased β-catenin and the phosphorylation levels of AKT and GSK-3β and decreased the expression of p21 in TE2 and TE5 cells. These results suggest that knockdown of NHE1 activates PI3K-AKT signaling in ESCC cells. Moreover, p53 status was different between TE2 cells and TE5 cells.p53 wasn’t expressed in TE2 cells, but was expressed in TE5 cells. Knockdown of NHE1 increased expression of p53 in TE5 cells (Figure 1E).Inhibition of apoptosis by knockdown of NHE1 was greater in TE5 cells than in TE2 cells (Figure 1D). These results suggest that p53 enhances inhibition of apoptosis by NHE1 siRNA in ESCC cells.

### NHE1 controls cell migration and invasion and affects molecular markers of EMT in ESCC cells

**Figure 2:**
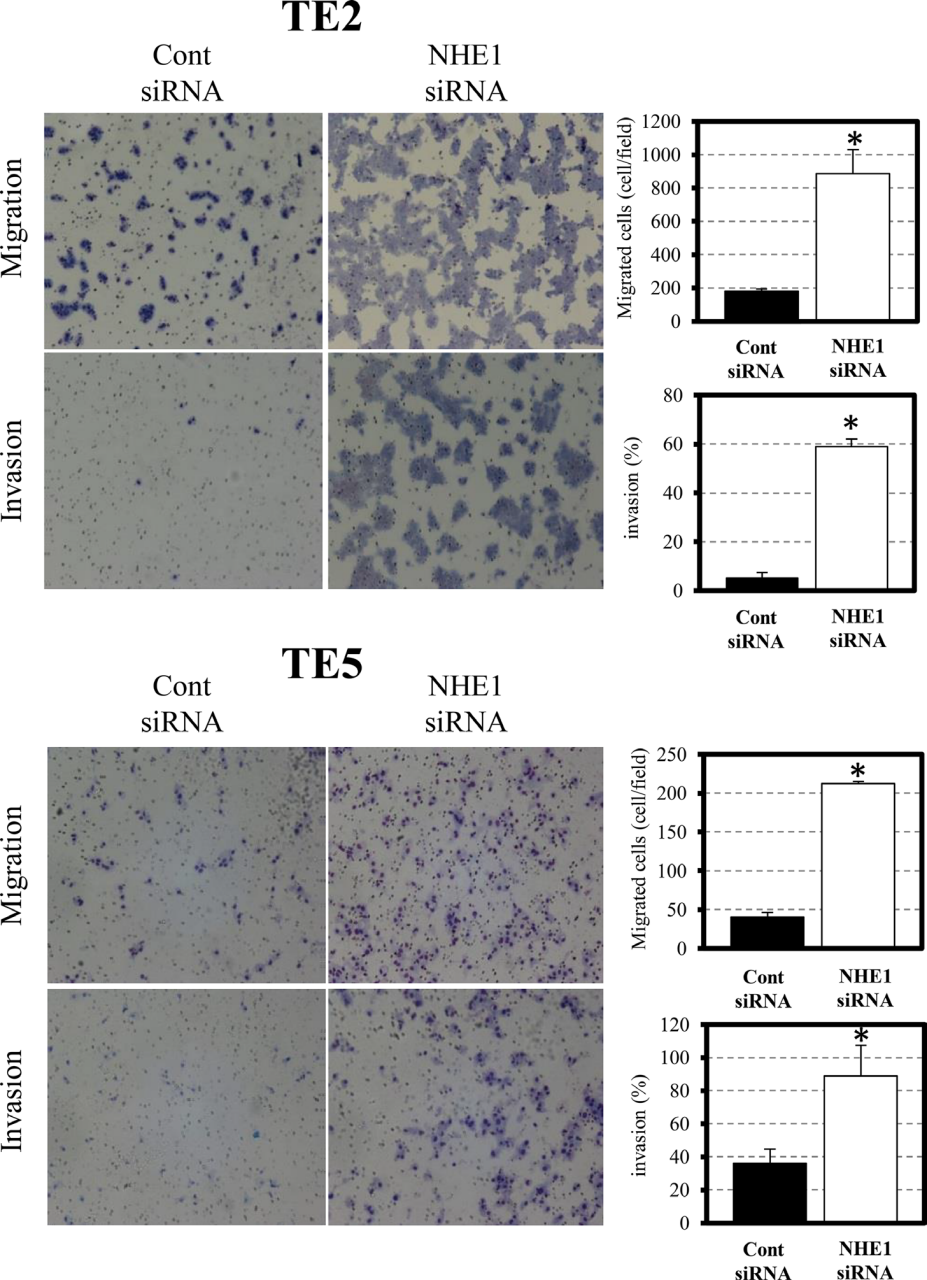
NHE1 controlled cell migration and invasion in ESCC cells The down-regulation of NHE1 significantly promoted cell migration and invasion in TE2 and TE5 cells. Cell migration and invasion were determined by the Boyden chamber assay. Mean ± SEM; *n* = 3. *P < 0.05 significantly different from the control siRNA group.

**Figure 3:**
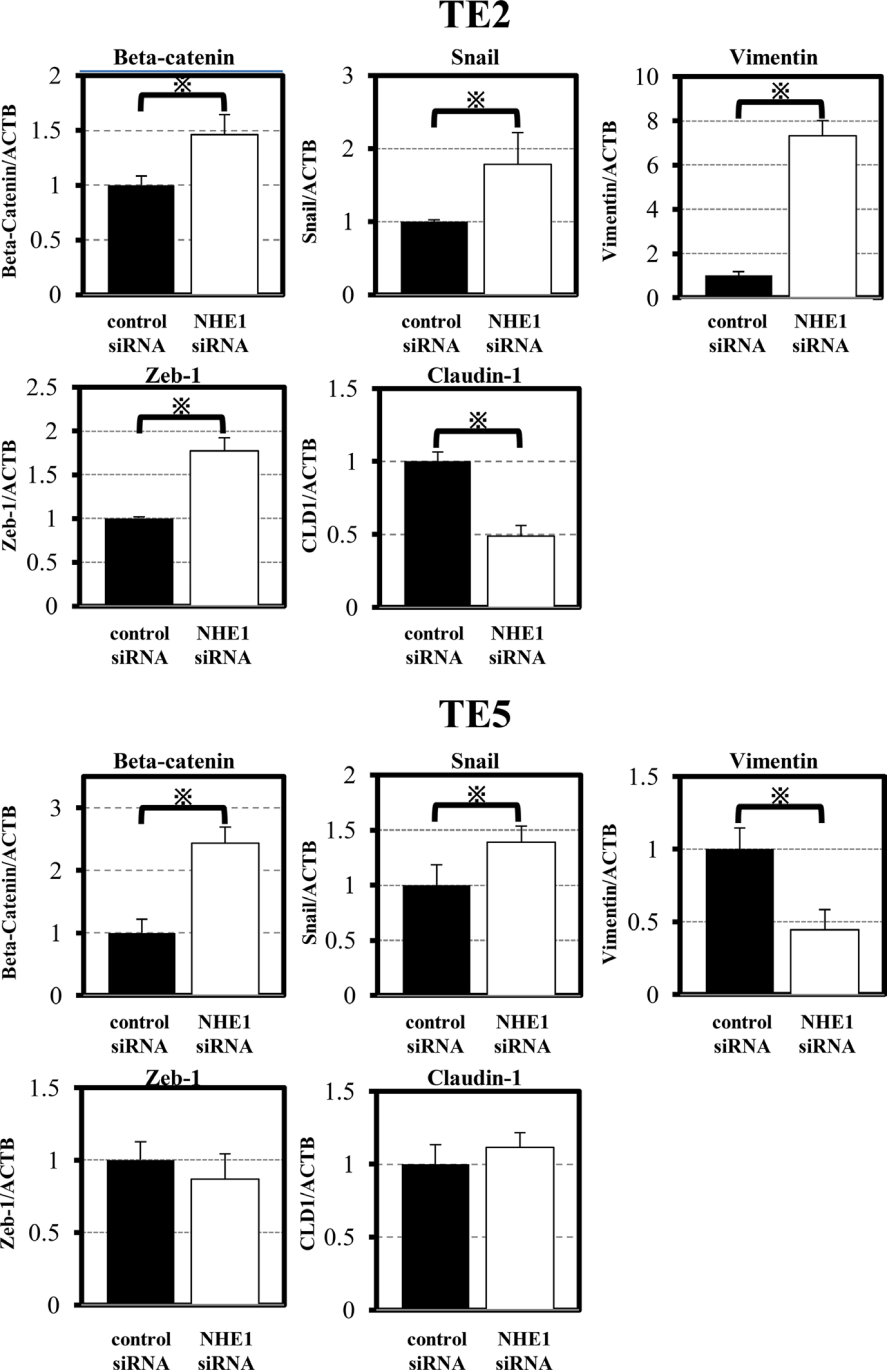
NHE1 regulated EMT markers in ESCC cells The down-regulation of NHE1 affected various EMT markers, particularly Snail and β-catenin. Mean ± SEM; *n* = 4. *P < 0.05 significantly different from the control siRNA group.

In TE2 and TE5 cells, the down-regulation of NHE1 significantly promoted cell migration and invasion (Figure 2). Since EMT has been implicated in cell invasion and cancer metastasis [27, 28], we evaluated changes in the levels of EMT markers by quantitative RT-PCR. The expression of Snail and β-catenin were up-regulated by the down-regulation of NHE1 in TE2 and TE5 cells (Figure 3). siNHE1 upregulated the expression of vimentin and Zeb-1 and down-regulated that of Claudin-1 in TE2 cells (Figure 3). These results indicated that downregulation of NHE1 promotes cell migration and invasion in ESCC cells by upregulating EMT markers, particularly Snail and β-catenin.

### Gene expression profiling in NHE1 siRNA-transfected cells

**Figure 4:**
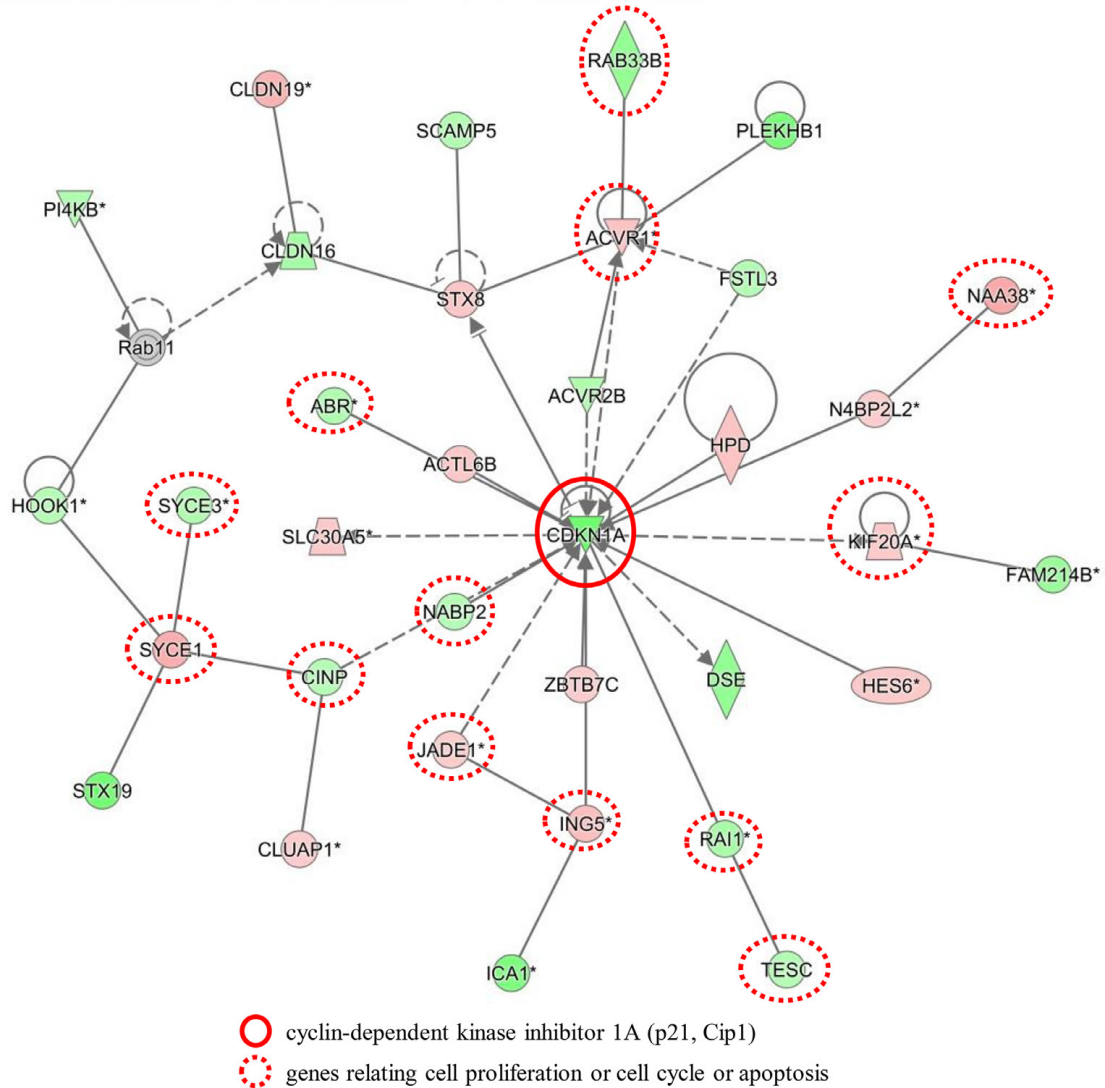
Network analyses by the ingenuity pathway analysis Top networks related to NHE1 knockdown according to the Ingenuity Pathway Analysis (Hematological Diseases, Hereditary Disorders and Metabolic Diseases).

We analyzed the gene expression profiles of NHE1-depleted TE2 cells in microarray and bioinformatic studies. The results of the microarray analysis showed that the expression levels of 6219 genes displayed fold changes of > 1.5 in TE2 cells following the depletion of NHE1. Of these genes, 2963 were up-regulated and 3256 were down-regulated in NHE1 siRNA-depleted TE2 cells. A list of 20 genes with expression levels that were the most strongly up- or down-regulated in NHE1-depleted TE2 cells is shown in Supplementary Table S1. An Ingenuity Pathway Analysis (IPA) showed that “Cancer” was the top-ranked disease and that “Cellular Movement”, “Cellular Development”, and “Cellular Growth and Proliferation” were some of the top-ranked biological functions related to the depletion of NHE1 (Supplementary Table S2). Furthermore, “Colorectal Cancer Metastasis Signaling” and “Regulation of the Epithelial-Mesenchymal Transition Pathway” were two of the top-ranked canonical pathways related to the depletion of NHE1 (Supplementary Table S3). IPA showed that the top-ranked network related to the knockdown of NHE1 was “Hematological Diseases, Hereditary Disorders, Metabolic Diseases” (Figure 4). This network included CDKN1A (p21, Cip1) and genes related to cell proliferation, the cell cycle, and apoptosis. These results indicated that the expression of NHE1 influences genes that regulate cellular growth, proliferation, apoptosis, metastasis, and EMT.

### Verification of gene expression by real-time quantitative RT-PCR

**Figure 5:**
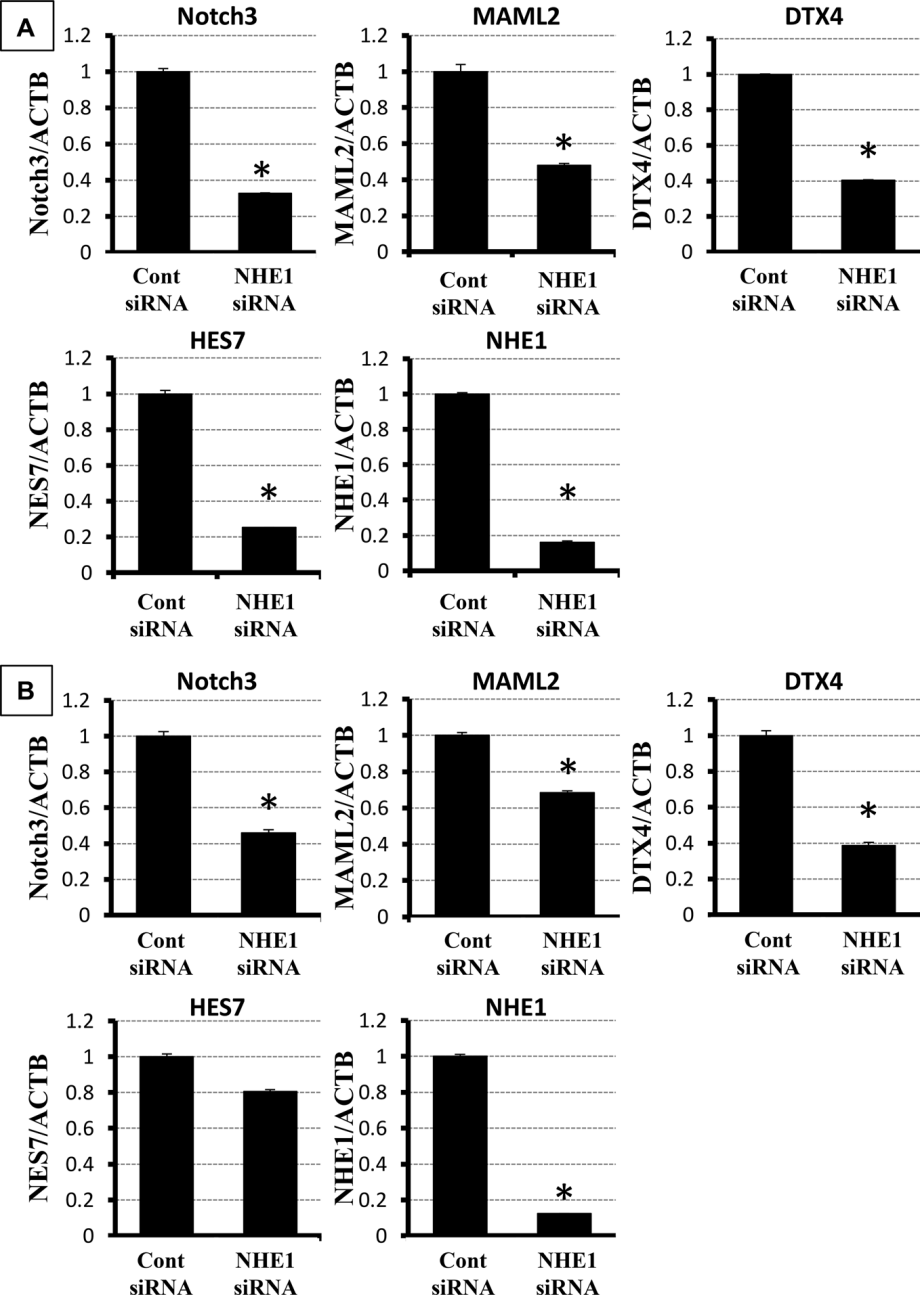
Verification of gene expression by real-time quantitative RT-PCR The expression of five selected genes (Notch3, MAML2, DTX4, HES7, and NHE1) was compared between NHE1 siRNA-transfected TE2 cells and control siRNA-transfected cells. The expression of each gene was normalized against ACTB. Mean ± SEM. *n* = 3. *P < 0.05 significantly different from the control siRNA group.

Notch signaling has been reported to regulate EMT in various cancer cells [29, 30]. The results of the microarray analysis also indicated that Notch signaling was down-regulated by the knockdown of NHE1 (Supplementary Figure S2). We selected five genes (Notch3, MAML2, DTX4, HES7, and NHE1) to confirm the results of the microarray analysis. Notch3, MAML2, DTX4, and HES7 were included in Notch signaling. The expression of the five genes was examined using quantitative RT-PCR. The expression levels of the five genes were significantly lower in NHE1-depleted TE2 cells than in control siRNA-transfected cells (Figure 5A). The same depletion of genes was confirmed in the TE5 cell line (Figure 5B). These results are consistent with the microarray results and suggest that knockdown of NHE1 suppresses Notch signaling in ESCC cells.

### NHE1 protein expression in human ESCC

**Figure 6:**
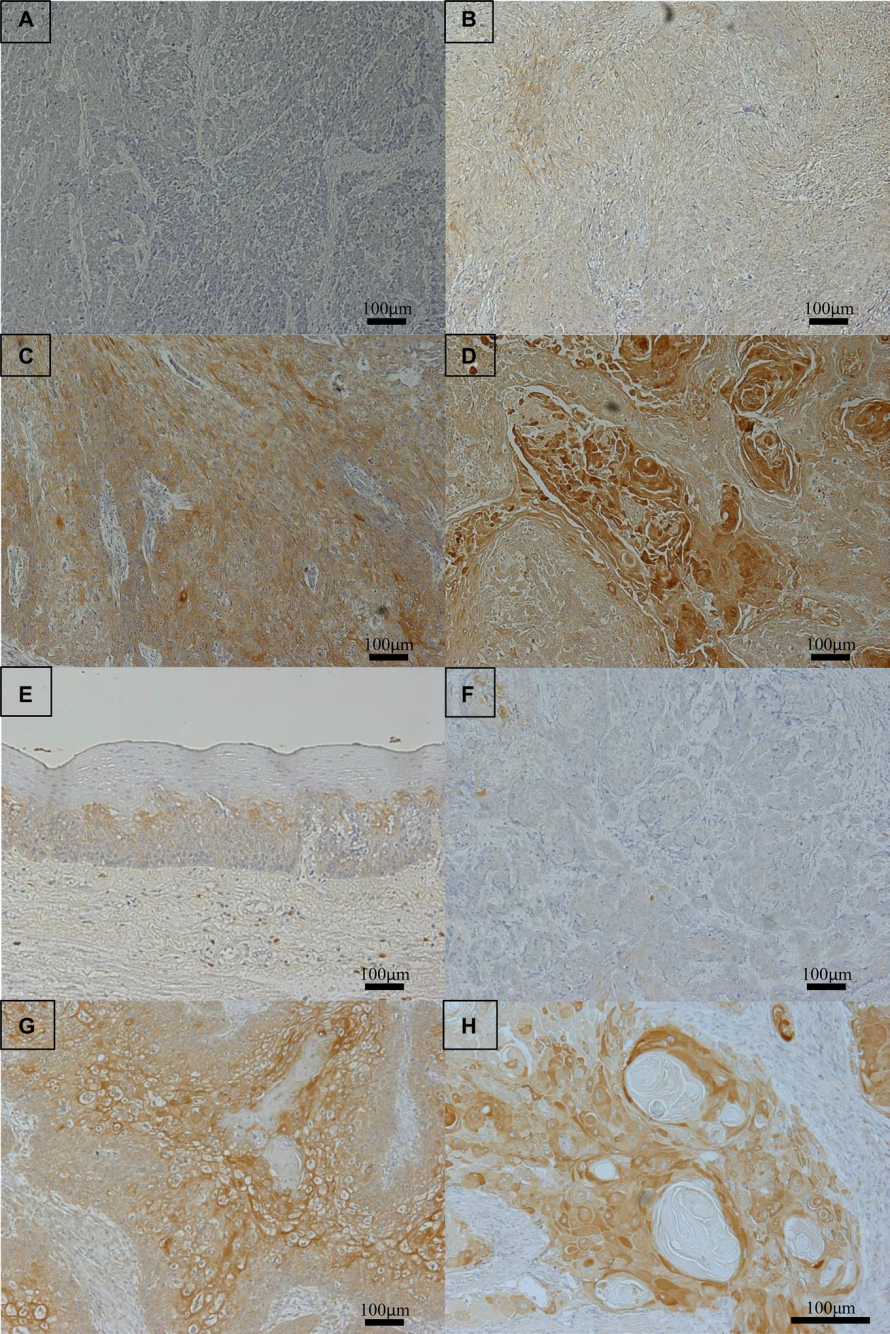
NHE1 protein expression in human esophageal squamous cell carcinoma (ESCC) (**A**–**D**). Photomicrographs of NHE1 immunohistochemistry are shown with the examples of score 0 (A), score 1 (B), score 2 (C), and score 3 (D). Magnification: ×100. (**E**) Immunohistochemical staining of non-cancerous esophageal epithelia with the NHE1 antibody. Magnification: ×100. (**F**) Immunohistochemical staining of primary human ESCC samples with the high grade expression of NHE1. Magnification: ×100. (**G**) Immunohistochemical staining of primary human ESCC samples with the low grade expression of NHE1. Magnification: ×100. (**H**) The high grade expression of NHE1 was observed around keratinization. Magnification: ×200.

**Figure 7:**
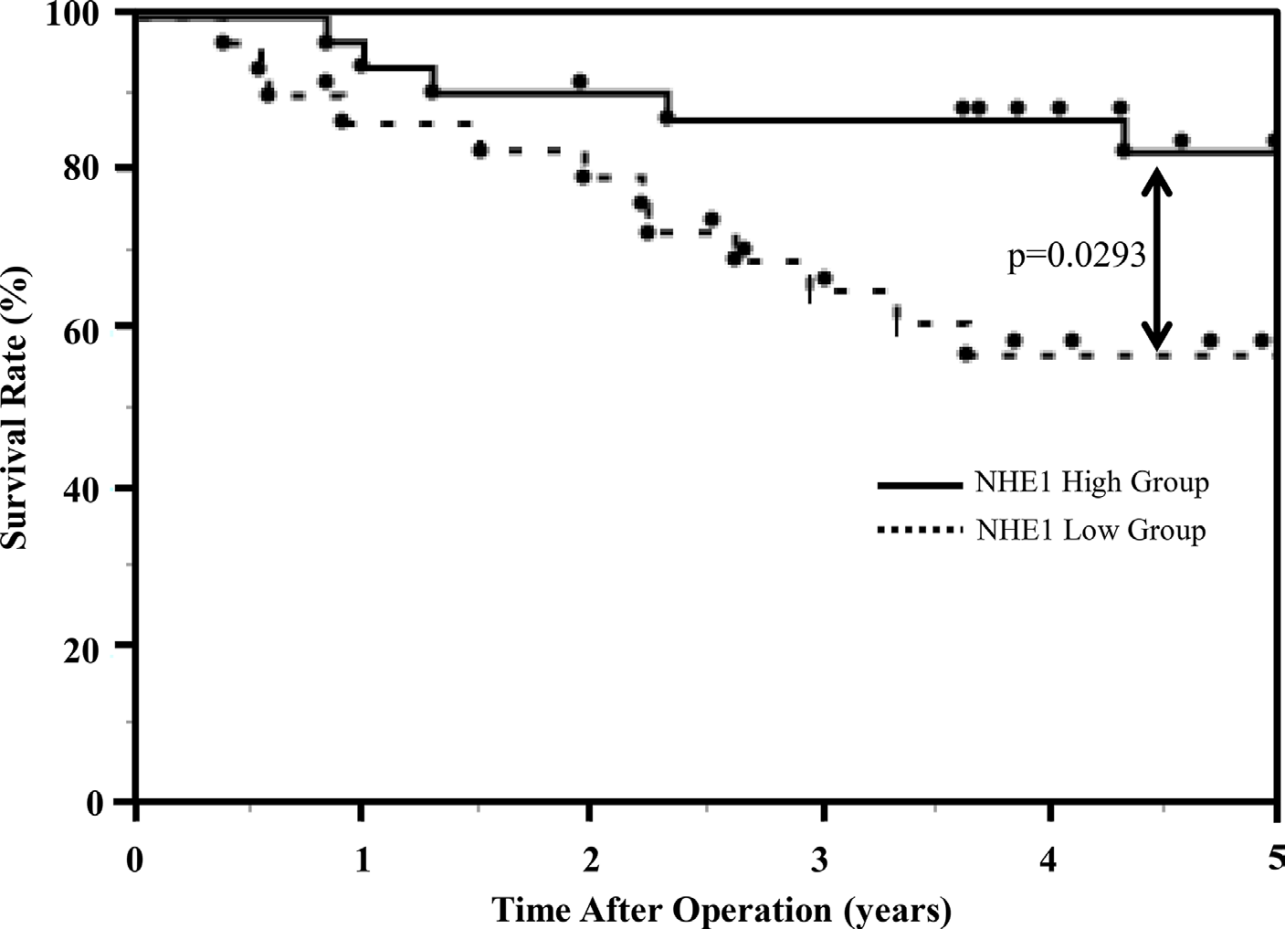
Survival curves of 61 ESCC patients. The 5-year overall survival rate was significantly poorer in the NHE1 low group than in the NHE1 high group (Log-rank test, *p* = 0.0293)

We further examined the expression of NHE1 in 61 primary tumor samples of human ESCC based on their immunohistochemical reactivities (Figure 6A–6D). The NHE1 protein was weakly expressed in non-cancerous esophageal epithelia (Figure 6E). The NHE1 protein was expressed in the plasma membrane and cytoplasm of carcinoma cells (Figure 6F, 6G). The strong expression of NHE1 was observed around keratinization (Figure 6H). On the contrary, the expression of Snail and β-catenin were weak in the part of high NHE1 expression (Supplement Figure 3).We compared two groups that were established based on the NHE1 staining scores described in the ‘‘Methods’’ section. Of the 61 patients, 30 (49%) were classified into the NHE1 low group and 31 (51%) into the NHE1 high group. The histological type correlated with the expression of NHE1 (Table 1), However, the expression of NHE1 did not correlate with other clinicopathological variables, including gender, age, lymphatic invasion, venous invasion, pathological depth of the tumor, or lymph node metastasis (Table 1). We also determined whether the expression of NHE1 was prognostic for ESCC patients after curative resection. The 5-year overall survival rate of the NHE1 low group was 57.0%, which was significantly poorer than that of the NHE1 high group (82.8%) (*p* = 0.029) (Figure 7). The univariate analysis showed that the presence of lymphatic invasion, lymph node metastasis, and pathological depth of the tumor correlated with a poor 5-year overall survival rate. A multivariate analysis with these four factors revealed that the presence of lymphatic invasion, pathological depth of the tumor and weak expression of NHE1 were independent prognostic factors (Table 2). Regarding the pattern of postoperative recurrence within 5 years, the number of patients with lymphogenous recurrence and hematogenous recurrence was significantly larger in the NHE1 low group than in the NHE1 high group (Table 3). These results suggest that the expression of NHE1 is induced in ESCC, and its stronger expression may be related to the good prognosis of patients with ESCC after curative resection.

**Table 1:** Association between clinicopathologic characteristics and NHE1 expression

Variable	NHE1 expression				*p* value
	low group(*n* = 30)		high group(*n* = 31)		
	*n*	(%)	*n*	(%)	
Gender					
Male	25	(83.3)	27	(87.1)	0.6784
Female	5	(16.7)	4	(12.9)	
Age					
<65	19	(63.3)	18	(58.1)	0.6735
≥65	11	(36.7)	13	(41.9)	
Location of Primary Tumor					
Ut	4	(13.3)	7	(22.6)	0.6372
Mt	17	(56.7)	16	(51.6)	
Lt	9	(30.0)	8	(25.8)	
Histological Type					
Well/moderately differentiated SCC	18	(60.0)	26	(83.9)	0.0356
Poorly differentiated SCC	12	(40.0)	5	(16.1)	
Lymphatic Invasion					
negative	16	(53.3)	12	(38.7)	0.2510
positive	14	(46.7)	19	(61.3)	
Venous Invasion					
negative	17	(56.7)	19	(61.3)	0.7135
positive	13	(43.3)	12	(38.7)	
INF					
INF a	8	(27.6)	3	(10.0)	0.0786
INF b–c	21	(72.4)	27	(90.0)	
pT					
pT1	13	(43.3)	14	(45.2)	0.8857
pT2–3	17	(56.7)	17	(54.8)	
pN					
pN0	13	(43.3)	14	(45.2)	0.8857
pN1–3	17	(56.7)	17	(54.8)	

**Table 2:** Univariate and multivariate analyses for prognostic factors associated with 5-year survival

Variable	*n*	Univariable		Multivariable		
		5-year survival rate (%)	*p* value	Risk Ratio	95 % CI	*p* value
Gender						
Male	52	76.2	0.6344			
Female	9	69.4				
Age						
< 65	37	68.4	0.7925			
≥65	24	73.2				
Histological Type						
Well/moderately differentiated SCC	44	73.1	0.4024			
Poorly differentiated SCC	17	63.7				
Lymphatic Invasion						
negative	33	80.5	0.0919			
positive	28	61.7				
Venous Invasion						
negative	36	81.5	0.0195	3.062	1.132–9.114	0.0273
positive	25	55.4				
pT						
pT1	27	87.6	0.004	4.505	1.444–19.771	0.0078
pT2–3	34	56.7				
pN						
pN0	27	84.7	0.0488	2.696	0.932–9.701	0.0680
pN1–3	34	59.7				
NHE1 expression						
high group	31	82.8	0.0293	3.570	1.291–11.484	0.0135
low group	30	57.0				

**Table 3:** Correlation between the pattern of postoperative recurrence within 5 years and NHE1 expression

	NHE1 low group (*n* = 30)		NHE1 high group (*n* =31)		*p* value
	*n*	%	*n*	%	
recurrence	18	60.0	6	19.4	0.0009
lymphogenous	11	36.7	2	6.5	0.0028
hematogenous	3	10.0	0	0	0.0355
focal	3	10.0	1	3.2	0.2757
dissemination	1	3.3	3	9.7	0.3060

## DISCUSSION

Recent studies have shown that ion channels and transporters in cancer cells play crucial roles in cell proliferation, migration, apoptosis, and differentiation [31, 32]. The expression of ion channels is altered in many primary human tumors and is regarded as a potential target for cancer therapy [33]. This is the first study to examine the expression of NHE1 in human ESCC tissue and the pathophysiological role of its expression in ESCC cells.

The phosphatidylinositol 3-kinase (PI3K)-AKT signaling pathway is activated in many cancers and triggers a cascade of responses that promote cell growth, proliferation, and survival [34, 35]. Therapeutic strategies that target PI3K–AKT signaling are considered to be promising in the treatment of cancer [36]. In normal esophageal epithelial cells, an inhibitor of NHE1 was shown to increase cytoprotective ROS generation, cell viability, and AKT phosphorylation under acid loading [37]. In our *in vitro* study, the knockdown of NHE1 promoted cell proliferation and inhibited apoptosis and also attenuated staurosporine stimulus-induced apoptosis. Furthermore, the down-regulation of NHE1 activated PI3K-AKT signaling in ESCC cells. These results indicate that NHE1 may exert suppressive effects on cell growth and malignancy through PI3K-AKT signaling in ESCC cells, and, thus, has potential as a new target for the treatment of ESCC.

Recent studies reported that EMT was involved in cancer progression and metastasis, and also that cancer cells undergoing EMT acquired stem cell-like properties and resistance to chemotherapy [27, 28]. Many signaling systems, such as the TGF-β, Notch, Wnt, and PI3K-AKT signal pathways, trigger EMT and crosstalk each other [28]. The expression of various EMT-related genes and proteins in tumors, including Snail, β-catenin, Twist, E-cadherin, and claudin 1, was previously reported to have prognostic value [38, 39]. Glycogen synthase kinase-3β (GSK-3β) facilitates the degeneration of Snail and β-catenin, while the activation of PI3K-AKT signaling phosphorylates GSK-3β and suppresses its function [40]. In the present study, we found that the knockdown of NHE1 in ESCC cells promoted cell migration and invasion and increased the expression of Snail and β-catenin. The results of the microarray analysis supported the depletion of NHE1 in TE2 cells inducing EMT transformation. The immunohistochemical investigation revealed that the expression of NHE1 in ESCC tissue samples correlated with 5-year survival rates and recurrence after esophagectomy. Taken together, these results suggest that suppression of NHE1 increases cell migration and invasion by promoting EMT transformation in ESCC cells and, thus, the expression of NHE1 in tissue samples may be a useful prognostic factor and predictor for metastasis.

The effects of NHE1 on EMT markers, such as Vimentin, ZEB-1 and Claudin-1, differ between TE2 cells and TE5 cells, and this might be because of the difference of characteristic between TE2 cells and TE5 cells. p53 status is different between TE2 cells and TE5 cells and p53 is upregulated by NHE1 knockdown in TE5 cells. p53 inhibits Snail and Zeb and suppresses EMT transformation by up-regulation of microRNAs [41]. Deletion of p53 upregulates expression of Fascin and vimentin via NF-κB signaling, and promotes cell invasion and migration in colorectal cancer cells [42]. Taken together, p53 status in ESCC cells may relieve EMT transformation by knockdown of NHE1.

The biological function of the Notch signaling pathway is critically context-dependent [43, 44]; however, previous studies reported that Notch signaling was activated in order to maintain stemness in the basal layer of the esophageal epithelia and exerted anti-oncogenic effects in ESCC [44–46]. Notch signaling modulates the expression of genes encoding proteins involved in tumor development, such as Snail, β-catenin, NF-κB, AKT, and p21 [47, 48]. In the esophageal squamous epithelium, Notch1 and Notch3 have been shown to be activated in terminal differentiation [49, 50]. Sato et al. reported that EGFR inhibitors upregulated Notch1, Notch3, and critical transcriptional factors in keratinocyte differentiation and suppressed TGF-β-induced EMT in the cancer stem-like cells of ESCC [46]. In the present study, the results of the microarray analysis revealed that the knockdown of NHE1 down-regulated Notch signaling in TE2 cells. We reconfirmed the results of the microarray analysis by verifying the expression of the 5 selected genes, and revealed that the expression levels of Notch3 and co-activators were significantly inhibited by the downregulation of NHE1.Expression of EMT markers, such as Snail, beta-catenin and Zeb-1, were increased by the down-regulation of NHE1. These results indicate that knockdown of NHE1 leads to EMT transformation through up-regulation of Snail, beta-catenin and other EMT markers by suppression of Notch signaling in ESCC cells.

In the present study, NHE1 was strongly expressed around keratinization, and the number of patients with undifferentiated histological type and patients with recurrence were significantly larger in the NHE1 low group than in the NHE1 high group. Knockdown of Notch3 in ESCC cells promotes EMT with the up-regulation of ZEB and impairs squamous differentiation mechanisms, leading to invasive growth and tumor cell dissemination [30]. Notch signaling maintains the differentiation in esophageal epithelia. These results indicate that strong NHE1 expression in ESCC tumor leads to well-differentiated carcinoma and less invasiveness via downregulation of Notch signaling.

Previous studies reported that the inhibition of NHE1 expression in cancer cells suppressed cell proliferation and invasion [18–20]. However, we herein found that the knockdown of NHE1 in ESCC cells promoted cell growth, invasion, and migration. NHE1 has been reported to have opposite effects on cell survival in cardiac myocytes and renal proximal tubule cells, which have been attributed to different stimuli for NHE1 activation and cell type specificity [51]. In renal proximal tubule cells, Na^+^ entry through NHE1 induces regulatory volume increase (RVI)-mediated defense against apoptotic stress. Furthermore, activation of NHE1 causes intracellular alkalization, which leads to inhibit BAD and caspases [51]. In myocardial cells, ischemic stimulation activates NHE1 and increases Na^+^ influx, which causes Ca^2+^ influx through Na^+^/Ca^2+^ exchanger and leads to cell death [52]. Na^+^/Ca^2+^ exchanger isn’t generally present in renal proximal tubule cells, and that is postulated to cause opposite role of NHE1 in renal tubule cells and myocardial cells. In normal esophageal epithelial cells, an inhibitor of NHE1 was found to exert protective effects against a low pH stimulation [37]. Lauritzen et al. showed that the inhibition of NHE1 sensitized ΔNErbB2-expressing breast cancer cells to cisplatin-induced death and reduced cell viability [53]. In contrast, Rebillard et al. found that the inhibition of NHE1 in human colon cancer cells reduced cisplatin-induced apoptosis triggered by the activation of ASMase and increases in membrane fluidity [21]. In the present study, the effects of the knockdown of NHE1 differed slightly between TE2 and TE5 cells. Taken together, the results of the present study suggest that NHE1 plays different roles, the detailed mechanisms of which remain unclear, in a manner that depends on the cell and cancer types.

In summary, we herein demonstrated that NHE1 plays a suppressive role in the proliferation, survival, migration, and invasion of ESCC cells, thereby abrogating the activation of the PI3K-AKT pathway and EMT transformation. The results of the microarray analysis also showed that NHE1 affected the expression of genes with functions related to EMT and the Notch signaling pathway. The results of the immunohistochemical examination revealed that the expression of NHE1 in human ESCC tissue was related to the histological type and rate of recurrence and served as a prognostic factor in ESCC patients. Although further investigations are necessary, our results suggest that NHE1 may be a useful biomarker for tumor development and a novel therapeutic target in the future treatment of ESCC.

## MATERIALS AND METHODS

### Cell culture and materials

The human ESCC cell lines TE2 and TE5 were obtained from the Cell Resource Center for Biomedical Research at the Institute of Development, Aging, and Cancer (Tohoku University, Sendai, Japan). These cells were grown in RPMI-1640 medium (Nacalai Tesque, Kyoto, Japan) supplemented with 100 U/mL of penicillin, 100 μg/mL of streptomycin, and 10% fetal bovine serum (FBS). Cells were cultured in flasks and dishes in a humidified incubator at 37°C under 5% CO_2_ in air. The polyclonal anti-NHE1 antibody used for the immunohistochemical analysis and protein assay was obtained from Santa Cruz Biotechnology (Santa Cruz, CA, USA). The following antibodies were used in the Western blot analysis; a monoclonal anti-AKT antibody, monoclonal anti-phospho AKT antibody, monoclonal anti-GSK-3β antibody, monoclonal anti-Phospho-GSK-3β antibody, polyclonal anti-β-catenin antibody, and monoclonal anti-p21 antibody were from Cell Signaling Technology, and a monoclonal anti-β-actin antibody and a monoclonal anti-p53 antibody were from Sigma-Aldrich (St. Louis, MO, USA). The following antibodies were used in the immunohistochemical staining; polyclonal anti-β-catenin antibody was from Cell Signaling Technology, and anti-Snail goat polyclonal antibody was from Abcam.

### Western blotting

Cells were harvested in M-PER lysis buffer (Pierce, Rockford, IL) supplemented with protease inhibitors (Pierce, Rockford, IL). Protein concentrations were measured with a modified Bradford assay (Bio-Rad, Hercules, CA). Cell lysates containing equal amounts of total protein were separated by SDS-PAGE and then transferred onto PVDF membranes (GE Healthcare, Piscataway, NJ). These membranes were then probed with the indicated antibodies, and proteins were detected using an ECL Plus Western Blotting Detection System (GE Healthcare, Piscataway, NJ).

### Small interfering RNA (siRNA) transfection

Cells were transfected with 10 nmol/L NHE1 siRNA (Stealth RNAi siRNA #HSS109889, Invitrogen, Carlsbad, CA) using the Lipofectamine RNAiMAX reagent (Invitrogen), according to the manufacturer's instructions. Medium containing siRNA was replaced with fresh medium after 24 h. Control siRNA (Stealth RNAi siRNA Negative Control; Invitrogen) was used as a negative control.

### Overexpression study

Control-HaloTag^®^ plasmid (Promega, G6591) and NHE1-HaloTag^®^ plasmid were transfected into TE2 cells and TE5 cells using FuGENE HD transfection reagents (Promega, E2311) following the manufacturer's instructions. Transfection of vector was confirmed by fluorescent microscopy for HaloTag^®^ fusion protein stained with the TMR conjugated HaloTag^®^ ligand (Promega, G8252) according to the manufacturer's protocol. Proliferation assay and apoptosis assay were conducted at 48 h after transfection.

### Cell proliferation

Cells were seeded on 6-well plates at a density of 1.0 × 10^5^ cells per well and incubated at 37°C with 5% CO_2_. siRNA was transfected 24 h after the cells had been seeded. Cells were detached from the flasks with trypsin-EDTA 72 h after siRNA transfection and were counted using a hemocytometer.

### Analysis of apoptotic cells

Cells were treated with staurosporine (200 nmol/L), which induced intrinsic apoptosis via the activation of caspase-3, for 24 h. Apoptotic cells were determined using an Annexin V-FITC kit (Beckman Coulter, Tokyo, Japan), which contained FITC-conjugated Annexin V and propidium iodide (PI), as directed by the manufacturer. Apoptotic cells were analyzed by flow cytometry with BD Accuri C6 (BD Biosciences, Tokyo, Japan).

### Real-time reverse transcription-polymerase chain reaction (RT-PCR)

Total RNA was extracted using an RNeasy kit (Qiagen, Valencia, CA). Messenger RNA (mRNA) expression was measured by quantitative real-time PCR (7300Real-Time PCR System; Applied Biosystems, Foster City, CA) with TaqMan Gene Expression Assays (Applied Biosystems), according to the manufacturer's instructions. Expression levels were measured for the following genes: NHE1 (Hs 00300047 m1), Snail (Hs 00195591 m1), β-catenin (Hs 00355049 m1), vimentin (Hs 00185584 m1), Zeb-1 (Hs 00232783 m1), claudin-1 (Hs 00221623 m1), Notch 1 (Hs 01062014 m1), DTX 4 (Hs 00302288 m1), HES 7 (Hs 00261517 m1), and MAML 2 (Hs 00418423 m1) (Applied Biosystems). Expression was normalized for the NHE1 gene to the housekeeping gene beta-actin (ACTB,Hs01060665 g1; Applied Biosystems). Assays were performed in duplicate.

### Analysis of cell migration and invasion

The migration assay was conducted using a Cell Culture Insert with a pore size of 8 μm (BD Biosciences, Bedford, MA). Biocoat Matrigel (BD Biosciences) was used to evaluate cell invasion potential. Briefly, cells (TE2: 2 × 10^5^ cells per well/ TE5: 5 × 10^5^ cells per well) were seeded on the upper chamber in serum-free medium 24 h after siRNA transfection. The lower chamber contained medium with 10% FBS. The chambers were incubated for the predetermined times (TE2:48 h/ TE5: 72 h) at 37°C with 5% CO_2_, and non-migrating or non-invading cells were removed from the upper side of the membrane by scrubbing with cotton swabs. Migrated or invaded cells were fixed on the membrane and stained with Diff-Quick staining reagents (Sysmex, Kobe, Japan). The migrated or invaded cells on the lower side of the membrane were counted in four independent fields of view at 100x magnification of each insert. Each assay was performed in triplicate.

### Microarray sample preparation and hybridization

total RNA was extracted using an RNeasy kit (Qiagen). RNA quality was monitored with an Agilent 2100 Bioanalyzer (Agilent Technologies, Santa Clara, CA). Cyanine-3 (Cy3)-labeled cRNA was prepared from 0.1 μg of total RNA using a Low Input Quick Amp Labeling Kit (Agilent), according to the manufacturer's instructions. Samples were purified using RNeasy columns (Qiagen). A total of 0.60 μg of Cy3-labeled cRNA was fragmented and hybridized to an Agilent SurePrint G3 Human Gene Expression 8 × 60 K ver 2.0 Microarray for 17 h. Slides were washed and scanned immediately on an Agilent DNA Microarray Scanner (G2565CA) using the one color scan setting for 8 × 60 K array slides.

### Processing of microarray data

Scanned images were analyzed with Feature Extraction Software 10.10 (Agilent) using default parameters to obtain background-subtracted and spatially detrended Processed Signal intensities. Signal transduction networks were analyzed with an Ingenuity Pathway Analysis (IPA) (Ingenuity Systems, Qiagen, Redwood City, CA).

### Patients and primary tissue samples

ESCC tumor samples were obtained from 61 patients with histologically confirmed primary ESCC who underwent esophagectomy at Kyoto Prefectural University of Medicine between 1999 and 2009 and were embedded in paraffin after 12 h of formalin fixation. Patient eligibility criteria were as follows: no synchronous or metachronous cancers (in addition to ESCC) and no preoperative chemotherapy or radiation therapy. We excluded patients with non-curative resected tumors or non-consecutive data. All patients provided written informed consent. Relevant clinicopathological and survival data were obtained from the hospital database. Staging was principally based on the International Union Against Cancer/tumor node metastasis Classification of Malignant Tumors (7th edition).

### Immunohistochemistry

Paraffin sections (3-μm-thick) of tumor tissues were subjected to immunohistochemical staining using the avidin-biotin-peroxidase method. Briefly, paraffin sections were dewaxed with xylene and hydrated with a graded series of alcohol. Endogenous peroxidases were quenched by incubating the sections for 30 min in 0.3% H_2_O_2_. Sections were then treated with a protein blocker and incubated at 4°C overnight with antibody. The avidin-biotin-peroxidase complex (Vectastain ABC Elite kit; Vector laboratories, Burlingame, CA) was visualized with diaminobenzidine tetrahydrochloride. Sections were counterstained with hematoxylin. These sections were then dehydrated through a graded series of alcohols, cleared in xylene, and mounted. Negative control sections were produced by omitting the primary antibody.

Immunohistochemical samples stained with NHE1 were graded semi-quantitatively based on the staining intensity and percentage of positive tumor cells. Staining intensity was scored as 0 (no staining, Figure 6A), 1 (weak staining, Figure 6B), 2 (moderate staining, Figure 6C), or 3 (strong staining, Figure 6D). Weak staining was observed in normal esophageal epithelia (Figure 6E), and moderate/strong staining was defined as NHE1-positive cells. The median proportion of NHE1-positive cells was 10%. The proportion of NHE1-positive cells > 10% was defined as high grade NHE1 expression (Figure 6G), and the proportion of NHE1-positive cells ≤ 10% was defined as low grade NHE1 expression (Figure 6F), respectively.

### Statistical analysis

Fisher's exact test was used to evaluate differences between proportions, and the Student's *t-test* was employed to evaluate continuous variables. Survival curves were constructed using the Kaplan-Meier method, and differences in survival were examined using the Log-rank test. A multivariate analysis of the factors influencing survival was performed using Cox's proportional hazard model. Differences were considered significant when the relevant *p value* was < 0.05. These analyses were performed using the statistical software JMP (version 8, SAS Institute Inc., Cary, NC).

## SUPPLEMENTARY MATERIALS FIGURES AND TABLES


