# Supplementary material for: Na+/H+ exchanger 1 has tumor suppressive activity and prognostic value in esophageal squamous cell carcinoma
Source: Oncotarget. 2016 Nov 26;8(2):2209–23. doi: 10.18632/oncotarget.13645 (PMC5356793; doi:10.18632/oncotarget.13645)
Supplement: Supplementary file 1 [file oncotarget-08-2209-s001.pdf]

Na^+/H^+ exchanger 1 has tumor suppressive activity and prognostic value in esophageal squamous cell carcinoma

Supplementary Materials

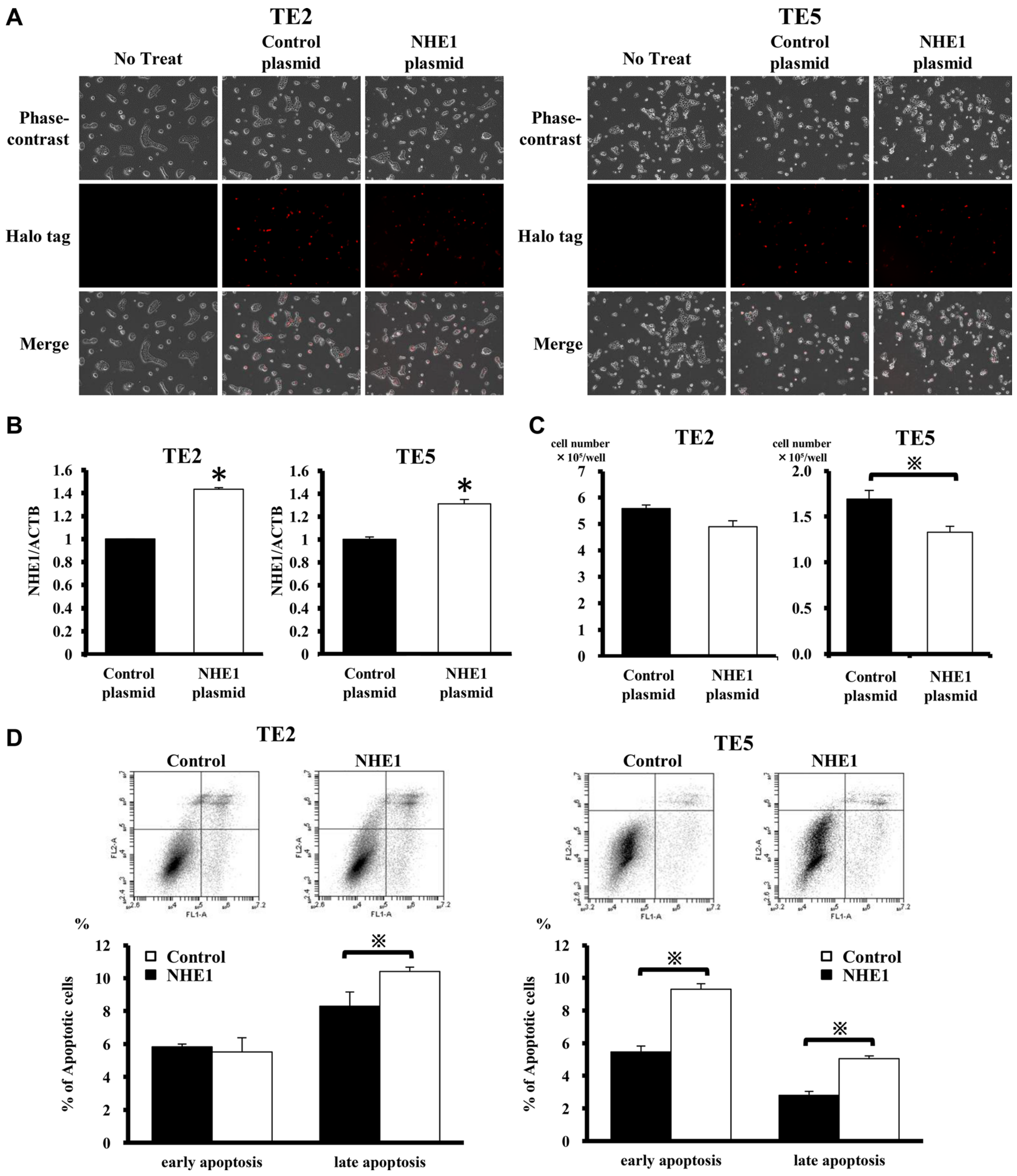

Supplementary Figure S1: NHE1overexpression inhibit cell proliferation of and induced apoptosis in ESCC cells. (**A**) Fluorescent microscopy for HaloTag® fusion protein. Magnification: ×10. (**B**) NHE1 plasmid increased NHE1 mRNA levels in TE2 and TE5 cells. Mean ± SEM; $n = 3$. *$P < 0.05$ significantly different from the control plasmid group. (**C**) The overexpression of NHE1 inhibited the proliferation of TE2 and TE5 cells. Mean ± SEM; $n = 3$. *$P < 0.05$ significantly different from the control plasmid group. (**D**) The overexpression of NHE1 induced apoptosis in TE2 and TE5 cells. Mean ± SEM. $n = 3$. *$P < 0.05$ significantly different from the control plasmid group.

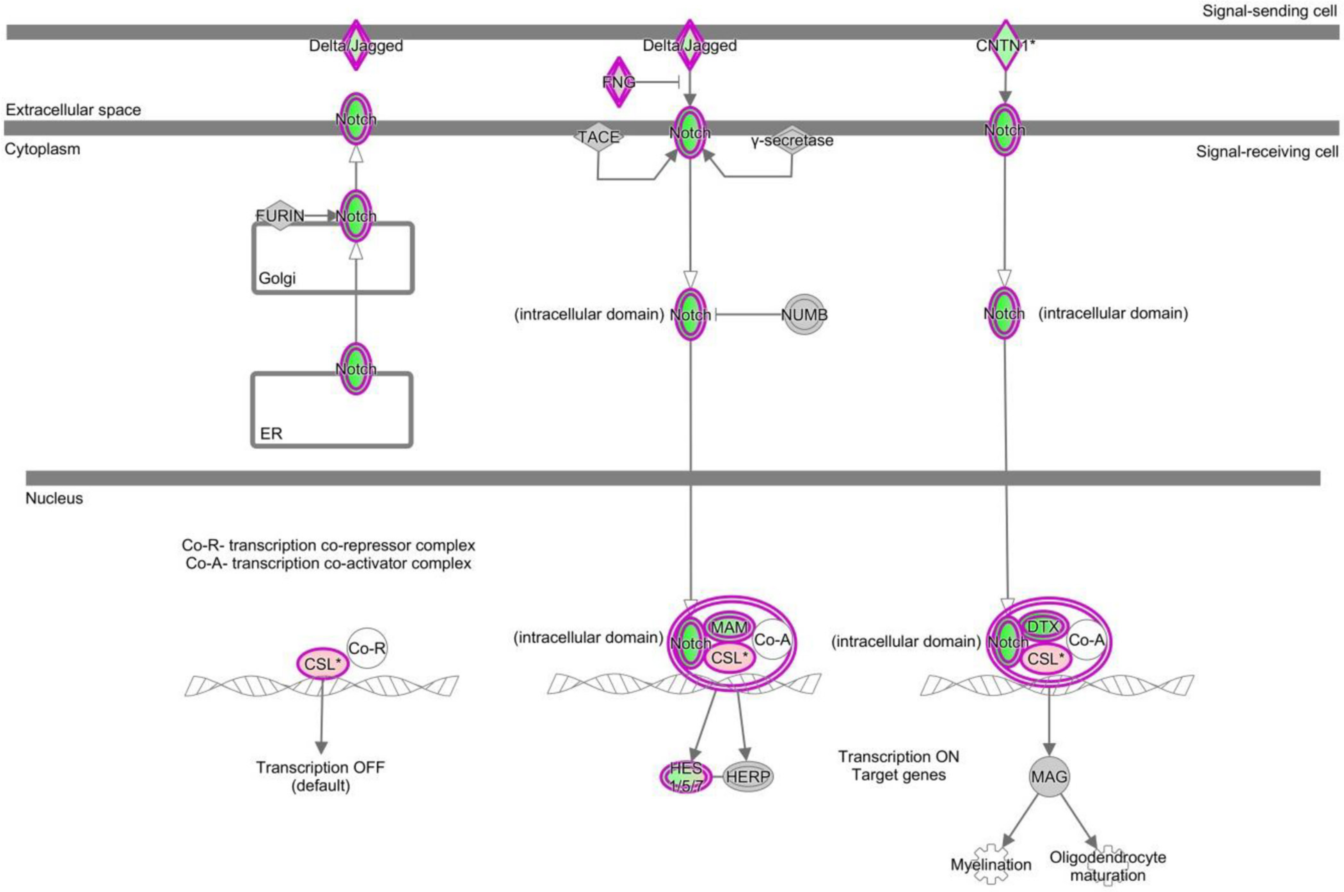

Supplementery Figure S2: Notch signaling was down-regulated in NHE1-depleted TE2 cells. Notch signaling was down-regulated. Factors which expression was reduced are shown in green.

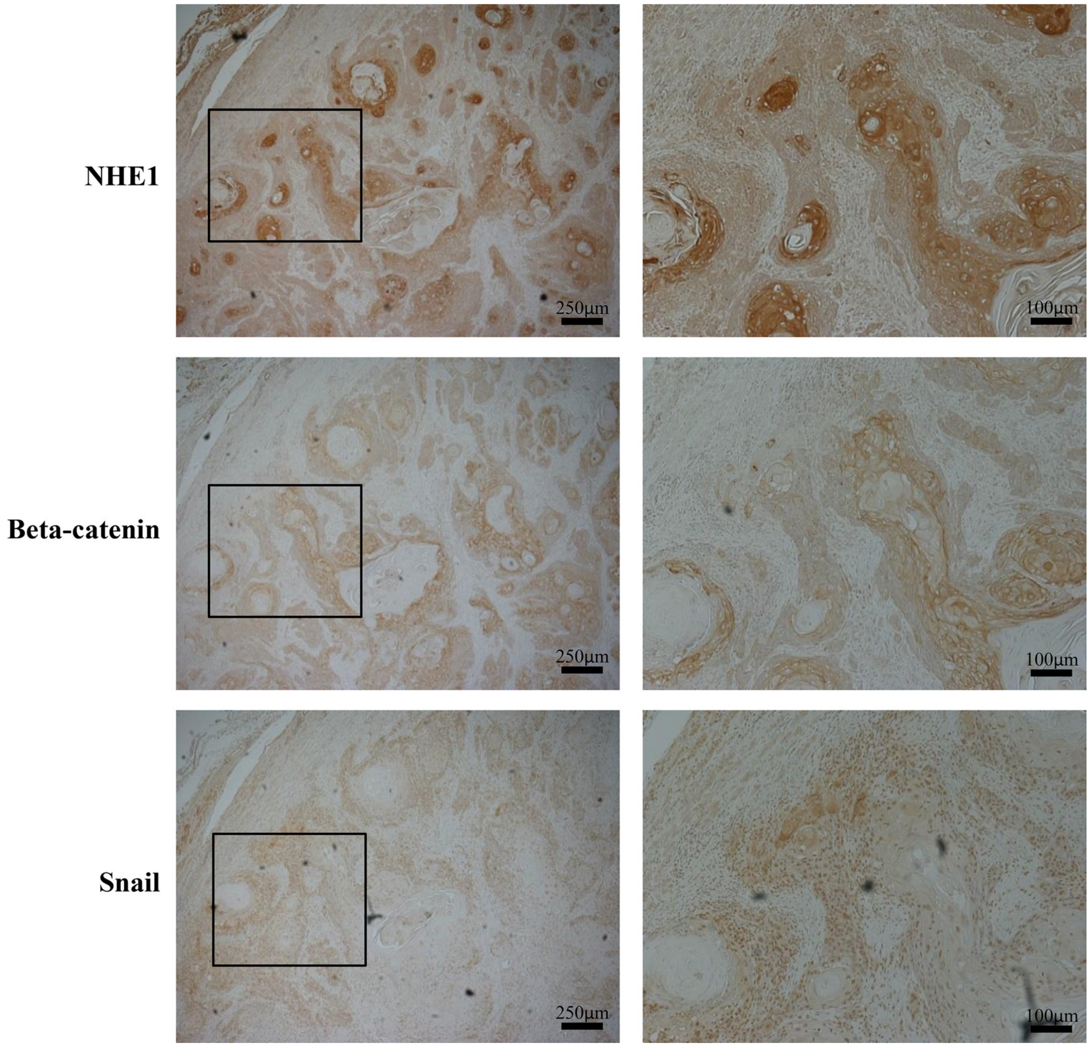

Supplementary Figure 3: Correlation between expression of NHE1 and expression of Snail and in human esophageal squamous cell carcinoma (ESCC). The expression of Snail and Beta-catenin was week in the part of high NHE1 expression. *Left*: Magnification: ×40. *Right*: enlarged view of left picture. Magnification: ×100.

Supplementary Table S1: The 20 genes that displayed the greatest changes in their expressions in the NHE1 siRNA transfected TE2 cells. See Supplementary_Table_S1

Supplementary Table S2: top diseases and biological functions related to NHE1 knockdown according to the ingenuity pathway analysis

Category	Name	Counts	*P* value
Diseases and Disorders	Cancer	1904	1.92E-04 - 2.31E-12
	Organismal Injury and Abnormalities	1954	1.94E-04 - 2.31E-12
	Reproductive System Disease	640	5.86E-05 - 2.31E-12
	Dermatological Diseases and Conditions	224	1.41E-04 - 2.54E-11
	Inflammatory Response	531	1.79E-04 - 1.36E-09
Molecular and Cellular Functions	Cellular Movement	612	2.02E-04 - 4.38E-14
	Cellular Development	935	2.01E-04 - 9.95E-11
	Lipid Metabolism	381	1.94E-04 - 1.39E-09
	Small Molecule Biochemistry	437	1.94E-04 - 1.39E-09
	Cellular Growth and Proliferation	908	1.79E-04 - 3.09E-09
Physiological System Development and Function	Embryonic Development	545	1.94E-04 - 7.94E-12
	Hair and Skin Development and Function	148	6.78E-05 - 7.94E-12
	Organ Development	513	1.94E-04 - 7.94E-12
	Organismal Development	835	2.02E-04 - 7.94E-12
	Tissue Development	818	1.94E-04 - 7.94E-12

Supplementary Table S3: Top canonical pathways and top networks related to NHE1 knockdown according to ingenuity pathway analysis

Category	Name	*P* value	count	ratio
Top canonical pathways				
	Axonal Guidance Signaling	1.94E-05	100/426	23.5 %
	Colorectal Cancer Metastasis Signaling	8.60E-05	59/231	25.5 %
	Regulation of the Epithelial-Mesenchymal Transition Pathway	1.08E-04	48/179	26.8 %
	Role of Osteoblasts, Osteoclasts and Chondrocytes in Rheumatoid Arthritis	1.23E-04	55/214	25.7 %
	Role of Macrophages, Fibroblasts and Endothelial Cells in Rheumatoid Arthritis	1.92E-04	68/283	24.0 %
Top networks			score	
	Hematological Disease, Hereditary Disorder, Metabolic Disease		35	
	Endocrine System Disorders, Organismal Injury and Abnormalities, Cell-To-Cell Signaling and Interaction		33	
	Developmental Disorder, Hereditary Disorder, Metabolic Disease		32	
	Auditory Disease, Hereditary Disorder, Neurological Disease		32	
	Cell Cycle, Developmental Disorder, Hereditary Disorder		32	
